# Levitated Micromagnets in Superconducting Traps: A New Platform for Tabletop Fundamental Physics Experiments

**DOI:** 10.3390/e24111642

**Published:** 2022-11-11

**Authors:** Andrea Vinante, Chris Timberlake, Hendrik Ulbricht

**Affiliations:** 1 CNR-Istituto di Fotonica e Nanotecnologie and Fondazione Bruno Kessler, Via Alla Cascata 56/C, 38123 Trento, Italy; 2 School of Physics and Astronomy, University of Southampton, Southampton SO17 1BJ, UK

**Keywords:** magnetic levitation, magnetometry, fundamental physics

## Abstract

Magnetically levitated microparticles have been proposed as mechanical sensors with extreme sensitivity. In particular, micromagnets levitated above a superconductor can achieve very low levels of dissipation and thermal noise. In this paper, we review recent initial experiments and discuss the potential for using these systems as sensors of magnetic fields and rotational motion, as well as possible applications to fundamental physics.

## 1. Introduction

Micro and nanoparticles can be levitated in a vacuum with a variety of techniques such as optical tweezers, Paul traps and magnetic levitation. The interest in these experimental platforms has steeply increased in recent years, with a number of scientists converging from the different fields of atomic physics, micro-nanomechanics, optomechanics and micromagnetism. This situation has led to the definition of a novel field of research named levitodynamics [1].

The most striking feature of levitated systems in a vacuum is the extremely low level of dissipation that these systems can achieve, which implies very low levels of thermal noise and decoherence. In particular, they present a small number of modes (typically three translational and three rotational), which are extremely decoupled from their internal environment. In this sense, levitated systems behave as extremely sensitive mechanical resonators. Moreover, the frequencies of the modes are often controlled by external potentials. This offers much better flexibility and tunability with respect to conventional resonators. In turn, this allows a large number of possible applications in various fields, such as ultra low noise sensing [2], accelerometry [3,4], gravimetry, magnetometry [5,6], fundamental physics [7], detection of dark matter or gravitational waves, tests of quantum mechanics [8], collapse models [9] or quantum gravity, non-equilibrium thermodynamics and material science.

In this paper, we consider a specific class of levitodynamical systems, namely ferromagnetic micro or nanoparticles levitated above a superconductor. While in the macroscopic domain, the levitation of magnets above superconductors (or vice versa) has been widely investigated, for instance, in the context of high-speed trains or inertial energy storage by rotating wheels, the levitation of micro and nanomagnets has been considered only recently. A first theoretical paper by Prat-Camps et al. [4] analyzed the dynamics of ferromagnets levitated above a punctured superconducting film and estimated the sensitivity to weak forces and accelerations. Later on, several pioneering experiments were performed, providing a first characterization of levitated ferromagnets under different levitation conditions, in particular, above type II [10,11] and type I [3,12] superconductors. Here, we review the current state and progress of this field. In Section 2, we analyze simple models of a levitated ferromagnet above a plane. In Section 3, we review published experiments and discuss potential improvements that we may expect in the near future. In Section 4, we will discuss, in more detail, the sensitivity of levitated magnets for specific applications, such as magnetic fields, torque, force sensing, as well as potential applications in the context of fundamental physics.

## 2. Theoretical Models

The basic mechanism allowing for the levitation of a hard ferromagnet above a superconductor is the repulsive interaction between the magnet and the surface currents in the superconductor. For a type-I superconductor in a well-shielded environment, the surface currents are entirely produced by the Meissner effect, which acts so as to completely shield the magnetic field produced by the magnet inside the superconductor. Since the Meissner effect has a positive energy cost [13], i.e., approaching the magnet to the superconductor requires positive work, the interaction between the magnet and the superconductor is always repulsive. This mechanism can be employed to float a magnet either inside a superconducting cavity or, in combination with gravity, above a superconducting surface. Similar considerations are valid for a type-II superconductor. Here, however, the levitation mechanism is complicated by the interaction with quantized flux vortices trapped in the superconductor.

Theoretically, the levitation mechanism cannot be modeled analytically unless for extremely simple situations. Full modeling requires solving the magnetostatic problem for the specific system and evaluating the magnet-superconductor interaction energy as a function of the position and orientation of the magnet with respect to the superconductor. Once this has been performed, the equilibrium configurations and small oscillation frequencies can be calculated. A general approach to solving the magnetostatic problem for an arbitrary system requires Finite Element Modeling (FEM) simulations.

Using FEM simulations, Prat-Camps et al. [4] analyzed a specific strategy to trap levitated magnetic spheres over a wide size range, from the nanoscale up to millimeter scale. The magnet was supposed to be levitated above an infinite type-I superconducting thin film in the presence of gravity. A circular hole in the film was used to confine the horizontal motion. The authors show that under a specific range of parameters, the levitation is stable in all degrees of freedom. Furthermore, they analyzed the limiting dissipating mechanisms theoretically, finding that this scheme can be used to realize ultrasensitive force sensors, with sensitivity down to $10^{-23}$ N/$\sqrt{\mathrm{Hz}}$ for magnets in the 100 nm range and accelerometers with sensitivity down to $10^{-14}$$g/\sqrt{\mathrm{Hz}}$ for magnets in the 10 mm range.

**Figure 1:**
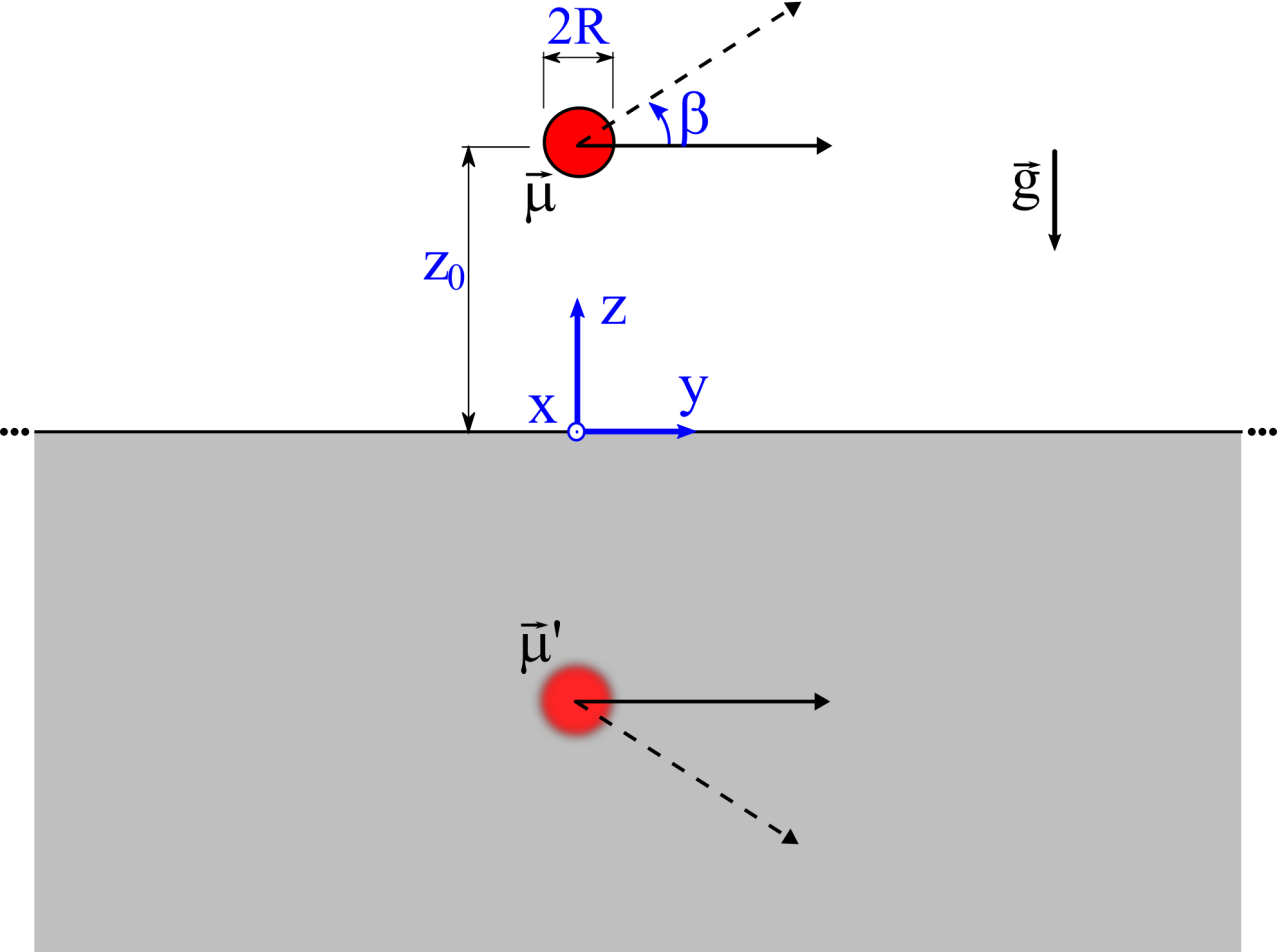
Image method to provide analytical solutions to the problem of a magnetic dipole above a superconducting plane. The magnetic dipole $\vec{\mu }$ is placed at a height $z_{0}$ above the infinite superconducting plane at z=0, with a librational angle β with respect to the x−y plane, in the presence of gravity acceleration $\vec{g}$. The field generated by the superconductor in the upper half of the space z>0 is equivalent to the field of a fictitious mirrored dipole ${\vec{\mu }}^{\prime }$ with the same magnitude. The force and potential energy of the real dipole can be derived accordingly.

However, for specific simple geometries of the superconductor, such as an infinite plane or a sphere, the magnetostatic problem can be solved analytically using the image method [14]. This allows more insight into the physics of levitation to be gained. For instance, let us consider a simple case, shown in Figure 1, with a magnetic dipole $\vec{\mu }\left(\vec{r}\right)$ above a superconducting plane, in the presence of gravity acceleration *g* [12]. Under these conditions, the horizontal motion is free, and the interaction energy between the magnetic dipole and the superconductor depends only on μ, the mass *m*, *g* and on two geometrical parameters: the height *z* of the dipole above the plane, and the angle β between the dipole and the horizontal plane. Although it may appear oversimplified, this model approximately reproduces the experiment performed by Vinante et al. [12], where the trap consisted of a cylindrical well in a bulk lead block.

According to the image method [14], the interaction energy between the magnet and the superconducting plane is one-half of the interaction energy with an image dipole $\vec{\mu^{\prime }}$ with the same magnetic moment placed at the mirrored position with mirrored orientation. This leads to:

(1) $$U=\frac{\mu_{0}\mu^{2}}{64\pi z^{3}}\left(1+\sin^{2}\beta \right)+mgz$$

where the gravitational energy is added to the magnetic one.

Energy minimization provides the equilibrium position $\beta =\beta_{0}=0$ and $z=z_{0}$, with:

(2) $$z_{0}={\left(\frac{3\mu_{0}\mu^{2}}{64\pi mg}\right)}^{\frac{1}{4}}.$$

Therefore, at equilibrium, the dipole lies horizontally and is free to move and rotate on the horizontal plane.

Instead, the motion along *z* and β is confined. The resonance frequencies of the translational *z* mode and the librational β mode can be calculated as $\omega_{z}=\sqrt{k_{z}/m}$ and $\omega_{\beta }=\sqrt{k_{\beta }/I}$, where *I* is the moment of inertia and $k_{z}$,$k_{\beta }$ are the spring constants:

(3) $$k_{z}=\frac{\partial^{2}U}{\partial z^{2}},$$

(4) $$k_{\beta }=\frac{\partial^{2}U}{\partial \beta^{2}}$$

evaluated at the equilibrium position.

Analytical expressions can be derived by further assuming that the particle is a homogeneous sphere with radius *R*, in which case:

(5) $$\omega_{z}=\sqrt{\frac{4g}{z_{0}}}$$

(6) $$\omega_{\beta }=\sqrt{\frac{5z_{0}g}{3R^{2}}}$$

**Figure 2:**
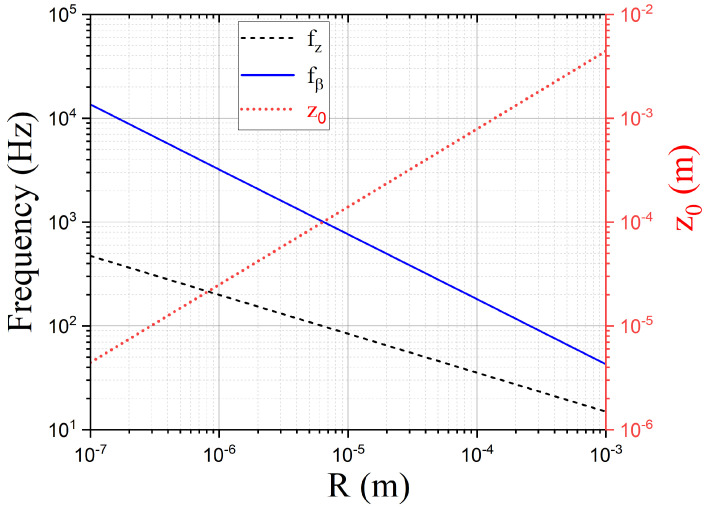
Resonance frequencies of the librational mode $f_{\beta }=\omega_{\beta }/2\pi$ (solid line) and vertical mode $f_{z}=\omega_{z}/2\pi$ (dashed line) for a microsphere of NdFeB (mass density ρ=7400 kg/m^3^, magnetization $M=6\times 10^{5}$ A/m) levitated above a superconducting plane, as a function of its radius *R*. The levitation height $z_{0}$ is shown on the same plot (dotted line and right axis).

Assuming uniform magnetization M=μ/V, where *V* is the sphere volume, the levitation height scales as $z_{0}\propto R^{3/4}$. This non-trivial dependence leads to $\omega_{z}\propto R^{-3/8}$ and $\omega_{\beta }\propto R^{-5/8}$. For typical NdFeB magnetic spheres with density ρ=7400 Kg/m^3^ and magnetization $M\approx 6\times 10^{5}$ A/m, the expected resonance frequencies, together with the levitation height $z_{0}$, are shown in Figure 2. We note that for the translational modes, the same dependence $\propto R^{-3/8}$ was found in ref. [4] for the case of a microsphere above a type-I superconducting film with a circular hole.

Another relevant parameter that can be derived from the above model is the maximum intensity of the magnetic field $B_{m}$ induced by the magnet at the superconductor surface at equilibrium conditions. One finds that $B_{m}\propto R^{3/4}$, with a corresponding effective magnitude, for the specific case of a NdFeB microsphere, to ∼10 mT for R=1 mm and ∼30 μT for R=10 μm. These values are well below the critical field of Pb ($B_{c}=80$ mT). However, for any given superconducting material, there will be a maximum size of a particle that can be levitated without exceeding the critical field.

In contrast to type-I superconductors, it is much more difficult to predict the resonant frequencies in the case of a type-II superconductor. Here, the dynamics are dominated by the interaction of the magnet with vortices. The density and distribution of vortices depends, on the external field and the magnet field at the time of the normal to superconducting transition. This can be partially accounted for by defining additional frozen images to model the frozen field from vortices [10]. Despite making the system less predictable, vortices can be very useful and convenient. For instance, they can be exploited to tune the resonance frequency of the levitated magnet. In fact, by varying the magnetic particle position before cooling through the superconducting transition, it is possible to tune the density of vortices and the strength of the interaction between particle and vortices, which sets the confinement and, ultimately, the resonant frequency.

Sources of dissipation in levitated magnets are also hard to predict, in general. Again, the scenario is, in principle, simpler in the case of type-I superconductors since the main source of dissipation, namely the motion of vortices, is absent. However, it has to be assessed experimentally if the conditions for the pure Meissner effect can be indeed met. The experiment by Vinante et al. [12] suggests that this is indeed the case for sufficient passive shielding from external fields. Furthermore, since the frequency is many orders of magnitude lower than the superconducting gap, intrinsic losses in the superconductor are likely negligible. Therefore, in addition to the unavoidable gas damping (which, in principle, can be reduced at will), the main sources of mechanical dissipation are likely located inside the levitated ferromagnet: eddy current losses if the material is also a conductor and ferromagnetic losses associated to hysteresis loops.

Eddy currents can be modeled quite accurately for a spherical geometry [12] if the electrical conductivity is known and under the assumption that the magnetization is saturated. For NdFeB microspheres with electrical conductivity of the order of $10^{6}$$\Omega^{-1}$ m^−1^, the eddy-current-limited *Q* factor is of the order of $10^{8}$ for radius R=1 mm for both *z* and β modes, and scales with $R^{21/8}$ and $R^{15/8}$, respectively, oming negligibly small at the micron scale.

By exclusion, ferromagnetic hysteresis losses related to domain walls are likely the limiting factor for the quality factor of multidomain micromagnets. Their effect depends on the effective dissipative part of the magnetic susceptibility of the material [12]. Quantitative estimation of this parameter requires a specific experimental investigation. However, for single-domain particles, where domain walls are absent, ferromagnetic losses are expected to be absent.

One can then speculate that for small particles in the single-domain limit, the effective magnetic dissipation can be extremely small, beyond the values measured in current experiments, which correspond to a maximum quality factor *Q*∼$10^{7}$ [12]. The extent to which the dissipation can be effectively reduced is still an important open question and needs to be experimentally assessed. In particular, when moving to the nanoscale, other types of dissipation, such as electrical non-contact losses, might become dominant.

Finally, it is worth pointing out that in the above modeling, we have tacitly assumed that the magnetization is rigidly attached to the easy magnetization axis of the magnet. This assumption implies completely neglecting the magnetization dynamics and is justified as long as the frequency of the mechanical motion is much lower than the magnetization dynamics. Indeed, for a levitated hard micromagnet, the ratio of magnetization to mechanical frequencies is in the order of $10^{10}$. Violations of this assumption may be associated with additional small dissipation contributions.

## 3. Review of Existing Experiments

Experiments can be broadly divided into two classes depending on the superconductor used for the trap, which can be type I or type II. In the latter case, the presence of vortices substantially changes the dynamical properties of the magnet.

### 3.1. Type-II Superconductors

A first attempt to levitate a micromagnet was reported by Wang et al. [10]. In this experiment, a PrFeB magnetic sphere with a diameter of 25 μm was levitated inside a cylindrical well machined in a bulk piece of niobium, and its motion was monitored optically by a CMOS camera. The frequencies of the translational modes, lying in the range 10–100 Hz, were monitored as a function of an externally applied magnetic field. The interpretation of the data was non-trivial due to flux-pinning in the niobium surface. A reasonable agreement was found with a model comprising a dynamical image dipole and a frozen image dipole. The former accounts for the Meissner repulsion, the latter for flux-pinning. The quality factor of the mechanical modes was in the range $10^{3}$–$10^{4}$, with a maximum value of $5\times 10^{4}$. The limiting dissipation was attributed to eddy currents in the magnetic particle itself, arising from the motion in the levitating and external magnetic field.

A second relevant experiment was reported by Gieseler et al. [11]. Here, NdFeB alloy magnetic microspheres with a radius of 15 and 23 μm were levitated above a YBCO film, which is a deeply type-II superconductor. A loading procedure employing a micromanipulator allowed precise positioning of the micromagnet upon cooldown of the superconductor. The final equilibrium position was determined by the flux-pinning of the magnetic field emanating from the micromagnet itself at the superconducting transition. In this way, it was possible to control the levitation height over the range of 2 to 7 times the particle radius. Frequencies of translational modes up to 3 kHz were measured, depending on the levitation height. Quality factors of order $10^{5}$ were measured, with a maximum recorded value of $1\times 10^{6}$. Subsequently, the micromagnet was coupled to a single NV center, using the fluorescence signal from the latter to detect the motion. The authors suggest the realistic possibility of simultaneously achieving a high mechanical Q factor, strong spin-mechanical coupling and cooperativity and long spin coherence. This makes spinning magnetomechanical systems a very promising platform for quantum sensing and for the exploration of novel quantum mesoscopic phenomena.

### 3.2. Type-I Superconductors

Type-I superconducting traps have been investigated for the first time by Timberlake et al. [3] by levitating a millimeter-sized NdFeB magnetic sphere above a ring of lead. The particle approached the ring by means of a micromanipulator. The authors proposed this system as a compact platform for ultrasensitive acceleration measurements using millimeter-scale levitated objects. Theoretically, the ring geometry allows stable trapping on three translational degrees of freedom [4]. The experiment has indeed demonstrated stable trapping in a high vacuum at liquid helium temperature. The particle motion was monitored by an optical readout and featured translational modes in the range 4–31 Hz. Furthermore, the authors measured a quality factor of $Q=5.5\times 10^{3}$, which was likely limited by eddy current losses in nearby metallic elements and in the conductive copper–nickel coating of the magnet. Despite the relatively low *Q* factor, a remarkably low acceleration noise of $1.2\times 10^{-10}$ g/$\sqrt{\mathrm{Hz}}$ was estimated in the thermal noise limit. Calibration measurements showed that the actual acceleration resolution was of the order of $10^{-7}$ g due to excess vibrational noise.

Subsequently, the same group developed a different strategy based on the Superconducting Quantum Interference Device (SQUID) readout. Since optical access was no longer needed, the whole setup could be fully enclosed inside a lead box, allowing for better shielding from external magnetic fields and reducing the potential effects of flux-pinning or mixed state. The trap consisted of a cylindrical well machined in the bottom part of the lead box. NdFeB alloy micromagnets with a diameter from 60 μm up to 1 mm were levitated in the well. The motion was monitored by a circular pick-up coil placed above the levitated micromagnet, connected to a remote dc SQUID. The combination of improved shielding and the absence of metallic parts in the vicinity of the micromagnet allowed very low dissipation and fairly good agreement with analytical and numerical predictions based on the full Meissner effect to be achieved, i.e., the model described in Section 2. In particular, all three translational modes and two librational modes were observed, with a frequency ranging from 5 up to 400 Hz. For some of the modes, clean ringdown measurements could be performed in vacuum at a resting gas pressure of ∼$10^{-6}$ mbar. Compared to all previous experiments, higher quality factors were achieved, in excess of $10^{7}$, with a damping rate down to $10^{-5}$ s^−1^, limited by residual gas damping. The ultimate limit of dissipation in an ultra-high vacuum could not be explored, but a residual dissipation contribution on top of gas damping was observed for the highest librational mode β. This dissipation was likely associated to ferromagnetic hysteresis losses in the micromagnet itself, while eddy currents were estimated to be negligible. In the same experiment, nonlinearities in the trapping potential associated with frequency shifts and crosscouplings were also explored.

The authors suggested several ways to exploit a low-frequency levitated micromagnet with ultra high quality factor for sensing applications, assuming it can be eventually operated at the thermal noise limit. In particular, ultrasensitive magnetometry has been identified as the most promising application. Several fundamental physics experiments were also proposed. These applications will be discussed in Section 4.

A third approach was recently reported by Raut et al. [15]. The authors levitated millimeter-sized neodymium magnets inside an aluminum microwave coaxial stub cavity. Here, two main novel features are in place. Aluminum is used instead of lead as the type-I superconductor, and the system is monitored by measuring the transmission spectrum of the cavity using a Vector Network Analyzer, so neither an optical nor SQUID readout is needed. A disadvantage of aluminum is that the critical temperature of 1.1 K requires sub kelvin refrigeration to achieve the levitation regime. On the other hand, the very long coherence length (∼1600 nm) and high ratio between coherence and penetration lengths (∼0.01) make aluminum probably the best possible material among type-I superconductors. Indeed, the authors report the observation of stable magnet levitation above the cavity stub, which is detected as a shift in the microwave cavity resonance. The levitation-induced shift is reproducible across different cooling cycles and in reasonable agreement with the shift calculated under the assumption of the full Meissner effect. The interesting conclusion is that the Meissner effect in aluminum is sufficient to levitate the magnet and fully expel the magnetic field, in spite of the fact that the initial local field at the surface of the magnet is larger than the critical field of aluminum. This suggests that aluminum is indeed a very good choice for levitation experiments. Furthermore, this setup appears promising as an optomechanical microwave readout could be implemented for the dynamical monitoring of the magnet motion. Moreover, it may be possible to couple the magnet to superconducting quantum devices. However, the authors have not reported a direct measurement of the mechanical motion so far.

### 3.3. Foreseen Near-Future Improvement and Challenges

The first experiments with levitating micromagnets above superconductors in a vacuum are very promising, but several improvements are needed in order to fully exploit this class of systems in ultrasensitive measurements.

First, a thorough investigation of the main dissipation mechanisms is needed to evaluate the ultimate limits of sensitivity. In particular, a wider investigation of different materials would be useful, both for the magnet and for the superconductor, including different material treatments. Then, one possible option is to move toward smaller magnets, possibly in the single domain limit, because these could show extremely low dissipation, with negligible contributions from eddy currents and internal magnetic losses. To levitate small magnets in the micron range, a chip approach will likely be needed. Loading the magnet in the trap could be an important issue.

The performance of levitated magnet sensors in the ultra-high vacuum limit has yet to be investigated. With a damping rate of the order of 1 μHz or less, two key improvements are mandatory: the implementation of feedback cooling in order to control all normal modes and efficient suppression of vibrational noise in the 1–1000 Hz region.

If the thermal noise limit can be achieved for mechanical modes with a damping rate lower than 1 μHz at T<1 K, extreme sensitivities will be achievable. For instance, a mm-radius magnet would achieve an acceleration noise in the order of $10^{-13}$ g/$\sqrt{\mathrm{Hz}}$, while a 10 μm-radius magnet would achieve a force noise in the order of $10^{-20}$ N/$\sqrt{\mathrm{Hz}}$. In the next section, we will analyze some possible applications of levitated micromagnet sensors.

## 4. Applications

### 4.1. Magnetometry and Torque Sensing

#### 4.1.1. Gyroscopic Regime

The most promising application of a levitated magnet with ultra-low mechanical noise is the realization of magnetometers with extreme magnetic field sensitivity. The first proposal in this direction was made by Jackson-Kimball et al. by analyzing the gyroscopic dynamics of a levitated ferromagnet [5]. This regime should be observable in the limit of a very weak external field, such that the induced Larmor precession angular momentum is much smaller than the intrinsic angular momentum due to the spin. This condition can be written as:

(7) $$\gamma B_{e}=\Omega_{L}\ll \Omega^{*}=\frac{N\hbar }{2I},$$

where $B_{e}$ is the external field, γ is the spin gyromagnetic ratio, $\Omega_{L}$ is the Larmor angular frequency, $\Omega^{*}$ is the critical angular precession, *N* is the total number of spins, proportional to the volume *V*, and *I* is the moment of inertia. For a magnet of ∼10 μm size, the critical frequency falls in the Hz range, and the corresponding field to nT or less.

In the gyroscopic regime, the dynamics are effectively the same as atomic spins in a magnetic field, allowing the estimation of the sensitivity in close analogy with atomic magnetometers. It was found that a ferromagnetic sensor can overcome the standard quantum limit (SQL) for independent spins by up to three orders of magnitude in magnetic field resolution. This can be naively explained by the fact that the magnet is a highly correlated system composed of a large number of spins locked in the direction of the macroscopic spin. At the same time, quantum noise in the transverse direction is rapidly averaged by internal ferromagnetic interactions, or in an alternate view; it is spread over a bandwidth (in order of hundreds of GHz as set by the ferromagnetic resonance) much larger than the precession frequency (order of Hz). Concrete calculations predict that a cylindrical magnetic needle with a length of 10 μm and a radius of 1 μm operated in a cryogenic vacuum (T=0.1 K, background gas density $10^{3}$ cm^−3^) can detect astonishingly weak magnetic fields down to $10^{-21}$ T, with practical ultimate limits set by gas collisions rather than by quantum noise.

Practical issues have, so far, prevented the observation of the gyroscopic regime in a micromagnet above a superconducting trap. In fact, in order to observe free precession on the horizontal plane, one needs the trapping potential to feature a perfect rotational symmetry. In type-II superconductors, this condition is practically impossible to achieve due to the unpredictable effect of flux pinning. In type I traps, other effects can show up. For instance, in ref. [12] the rotational symmetry was broken by finite tilts of the trap with respect to gravity. Further work is needed to control and suppress all rotational symmetry-breaking mechanisms, either by proper trap design or by active methods.

Under the assumption of perfect rotational symmetry, Fadeev et al. [7] have analyzed the dynamics of a levitated magnet above a type-I superconducting plane. The authors found that gyroscopic dynamics can indeed be observed, but with significant modifications with respect to the free-fall case. In particular, the image field $B_{i}$ produced by the superconductor results in an additional restoring torque for small librations off the horizontal position (β motion in Section 2) with respect to a system levitated without magnetic fields. The Larmor precession frequency case is then suppressed to:

(8) $$\Omega_{L}^{\prime }=\frac{\Omega_{L}}{1+\frac{\gamma B_{i}}{\Omega^{*}}}.$$

At the same time, the maximum external field that can be measured in the precessional regime is increased by the same factor:

(9) $$B_{\max}=\frac{\Omega^{*}}{\gamma }\left(1+\frac{\gamma B_{i}}{\Omega^{*}}\right)\approx B_{i}.$$

This situation, which combines the suppression of signal and increase in dynamics, closely resembles that of negative feedback.

The authors go further with their analysis by showing that the sensitivity to an externally applied magnetic field is preserved, in particular, the system can overcome the SQL on spin-based magnetometry. This makes the system particularly interesting for the detection of exotic spin–spin interactions between electrons. The latter is predicted by a broad class of theories beyond the standard model, including, for instance, axion-like particles, and would manifest as pseudomagnetic fields acting between spins. A ferromagnetic sensor in a superconducting trap would exploit two key features essential for the detection of these exotic interactions: the extreme sensitivity to magnetic or pseudomagnetic fields of the spin sensor and the inherent strong shielding from true magnetic interactions provided by the superconducting trap, assuming that the spin source is placed outside the trap. Improvements by more than two orders of magnitude over the current best limits on exotic spin–spin interactions have been predicted.

#### 4.1.2. Librational Regime

More recently, Vinante et al. [6] analyzed the performance of a torque-based ferromagnetic magnetometer in the more commonly observed librational regime. Here, the gyroscopic effects due to the intrinsic spins are negligible, and the micromagnet will simply librate around an equilibrium orientation determined by the total effective magnetic field, which may include an external field. Two librational modes will exist around two axes (say, x,y) orthogonal to the equilibrium direction (*z*).

The two librational modes can be simply modeled as effective harmonic oscillators in the respective angular coordinates. Evaluation of the torque resolution requires estimating the noise sources acting on these harmonic oscillators. One limiting factor is the thermal torque noise. Its power spectral density is given by the fluctuation-dissipation formula in the classical limit:

(10) $$S_{\tau_{T}}=4k_{B}T\frac{I\omega_{0}}{Q},$$

where *T* is the temperature, $k_{B}$ is the Boltzmann constant, *I* is the moment of inertia, $\omega_{0}$ is the resonance angular frequency and *Q* is the quality factor. The second limiting factor is the readout noise. Unless very challenging quantum nondemolition strategies are implemented, the lowest possible readout noise allowed by quantum mechanics is given by the standard quantum limit (SQL). In strict analogy to the SQL on force detection [16], the SQL on torque detection is given by:

(11) $$S_{\tau_{SQL}}=2\hbar I{\left[{\left(-\omega^{2}+\omega_{0}^{2}\right)}^{2}+{\left(\frac{\omega \omega_{0}}{Q}\right)}^{2}\right]}^{1/2}.$$

Equations (10) and (11) allow the estimation of the best torque resolution achievable as a function of the experimental parameters. The magnetic field resolution referred to an optimally oriented external magnetic field, inducing a torque τ=μB (where μ is the magnetic moment) is then given by $S_{B}=S_{\tau }/\mu^{2}$. Using this approach, Vinante et al. [6] showed that remarkably low magnetic field noise can be achieved. In order to compare it with other types of magnetometers, the authors used the so-called energy resolution as a relevant parameter, expressed by $E=S_{B}/\left(2\mu_{0}V\right)$, where *V* is the volume sampled by the sensor. Essentially, *E* represents the magnetic field resolution for a given sampled volume and a given measurement time. Most existing magnetometers, such as SQUIDs and atomic magnetometers, satisfy the relation E>ℏ, a sort of practical quantum limit better known as Energy Resolution Limit (ERL) [17]. The ERL has been substantially beaten only recently by a Bose-Einstein Condensate (BEC) based magnetometer [18]. According to ref. [6], a torque magnetometer based on a levitated magnet can beat ERL by up to 2–3 orders of magnitude in field amplitude, therefore, outperforming any existing or foreseen magnetometer. Concretely, for a levitated magnet of 30 μm, the ERL corresponds to $5\times 10^{-14}$ T/$\sqrt{\mathrm{Hz}}$, while the noise of a torque magnetometer would be about $1\times 10^{-16}$ T/$\sqrt{\mathrm{Hz}}$ in the thermal limit at T=4.2 K and $1\times 10^{-18}$ T/$\sqrt{\mathrm{Hz}}$ in the quantum limit. As in the gyroscopic case, a natural application would be the probing of exotic spin–spin interactions between electrons, with expected improvements of three orders of magnitude with respect to the existing limits.

### 4.2. Fundamental Physics

Here, we briefly discuss the potential of magnetic levitated particles amongst other large-mass mechanical systems for testing fundamental physics. We do not explain each idea in much detail, but rather summarize proposals published on the topic without attempting to be complete or inclusive.

The main reason why magnetic levitation is a strong contender as a candidate for an experimental test is that many noise and, indeed, decoherence sources are intrinsically reduced: (a) the trap is passive and has no externally controlled driving fields, which are an inherent source of noise when it comes to ultimate experiments, (b) the experiments are per definition at cryogenic conditions as the Meissner levitation works best for type-I superconductors. Low temperatures reduce thermal noises and if the experiment is cleverly designed, also brings extremely high vacuum (XHV). A disadvantage comes from the fact that acting on the system by external fields is much harder than in other trapping types, as the Meissner trap is passive—the trap is generated by the magnetic field of the particle itself in the diamagnetically induced interaction with the superconductor—and purposely shielded against external fields and the interaction of any electromagnetic origin, which could spoil the high-quality factor. In contrast, optical dipole or Paul ion traps make use of applied fields, which need to be actively controlled to form a stable trap, which always comes for the price of inducing noises but with the advantage that fine control of those trapping fields allows for more freedom in actively controlling the motion of the trapped particle and for motional state preparation, including squeezing [19]. Therefore, any experiments that rely on state active operation or driving of the motion in the Meissner trap have to be designed very well.

Besides the mature science and technology of cold atoms, there is a growing number of large-mass experiments designed to test different aspects of fundamental physics, such as tests of the quantum superposition principle or gravitationally induced decoherence, investigations of the interplay between general relativity and quantum mechanics, as well as the search for dark matter and dark energy. The experiments can be divided into two main classes: non-interferometric opto/electro/magneto-mechanical systems [20] and matter–wave interferometry with massive molecules or nanoparticles [21]. Large-mass systems push the envelope of realization of quantum states toward the macroscopic domain while providing an ultrasensitive test bed for standard models and exotic forces and acceleration. In analogy with cold atoms, the goal is to achieve quantum control of the center of mass motion, and many ideas for fundamental tests with atoms can be adapted to heavier particles; see ref. [22], which contains a comprehensive summary of fundamental physics to be tested by both atomic and large-mass systems.

#### 4.2.1. Non-Interferometric Experiments

Proposals for testing quantum mechanics, gravity and particle physics beyond the standard model based on the mature experimental platform of levitated optomechanics have been put forward and can be extended to magnetically levitated particles while highlighting the benefits of the low-noise cryogenic environment. Such proposals include the testing of predictions of General Relativity, e.g., high-frequency gravitational waves complementary to large-scale experiments, such as LIGO, VIRGO and the planned space-based instrument LISA, and are based on compact designs and geometries on the tabletop [23,24]. Proposals further include using levitated large-mass systems for the testing of classical gravity and space-time curvature [25], while prototype experiments demonstrating the technical capability of levitated systems have already been realized [26]. Another potential direction is toward probing the high-energy particle physics sector beyond the standard model, in particular, searching for dark matter [27,28] as well as dark energy [29] candidates. For instance, levitated magnetic sensors appear promising to test models predicting pseudo-magnetic fields. Exotic spin–spin interactions between electrons are one notable example [6,7].

Ideas for testing gravitationally interacting systems include proposals for testing the gravitational field generated by a massive quantum system [30,31] and for probing relativistic frame-dragging effects [32]. Finally, non-interferometric tests of the quantum superposition principle have already been demonstrated using low-temperature mechanical resonators [33], leading to significant experimental bounds on wave function collapse models. Due to lower thermomechanical noise, magnetically levitated systems have the potential for further improvements.

#### 4.2.2. Interferometric Experiments

Large-mass matter–wave interferometers can potentially test dark matter candidates [27,34,35,36], as well as the quantum superposition principle in the large-mass limit. Several designs of matter wave interferometers with nanoparticles have been theoretically proposed [37,38,39] employing different implementations of coherent beam splitters. A recent idea is to utilize the rotational degrees of freedom to test quantum mechanics at the macroscopic limit [40,41]. A key requirement of all these experiments is the availability of extended periods of time for the free evolution of the wave function, which could be provided by the super-clean environment of magnetic Meissner levitation. A more detailed discussion of experimental proposals for interferometric experiments with large-mass particles is reported in a recent review [42]. Magnetic levitation is one contender.

#### 4.2.3. New Ideas to Test the Interplay between Gravity and Quantum Mechanics

Several proposals have been put forward to investigate gravitational decoherence and semi-classical gravity [43,44], gravity-induced collapse of the wave function according to the Diosi-Penrose ideas [45,46,47,48], as well as stochastic gravity [49,50,51,52]. Ideas that have attracted much attention include the test of the quantumness of gravity by means of quantum information protocols applied to large-mass systems [53,54,55,56]. Further ideas are related to gravitational decoherence and general relativistic time dilation effects in interferometric settings [57]. A scientific debate is underway to explore the correct physics description and solid predictions of the effects [58,59]. Furthermore, experiments have been proposed to test quantum mechanics in accelerated reference frames by means of strongly accelerated tabletop systems. First investigations have been performed in research laboratories with quantum states of light, such as entangled states [60] or with systems showing other strong and non-classical correlations [61]. Potential extensions to large-mass systems can be envisioned [62].

#### 4.2.4. Testing Modified Gravity as an Alternative to Dark Matter

Modifications to Newton’s second law of dynamics F=ma, especially in the regime of low accelerations such as in the MOdified Newtonian Dynamics (MOND) theory [63], have been proposed as valid alternatives to dark matter [64] to explain, among other observables, the flat rotation curves of galaxies. In the astrophysical community, MOND is considered a viable approach to reproduce a number of galactic observations [65]. Recently, Milgrom has claimed that MOND can reproduce the full scaling of the angular momentum of disc galaxies as a function of galaxy mass [66]. MOND dynamics is fully equivalent to a Newtonian one above a characteristic acceleration $a_{0}=1.2\times 10^{-10}$ m/s^2^, but it significantly diverges at lower accelerations. Accurate measurements of the restoring torque of torsion pendulums have confirmed Newton’s second law down to $10^{-14}$ m/s^2^ [67], while gravity-based measurements have provided tests down to $10^{-12}$ m/s^2^ [68]. To fully test MOND, gravitational accelerations must be utilized. Klein has recently argued that these torsion pendulum experiments are actually compatible with MOND [69]. This state of affairs calls for further experimental investigations of MOND and MOND-like theories, possibly via experimental approaches different from torsion pendulums.

Given the high sensitivity of magnetically levitated particles, a concrete proposal to test MOND with this type of setup has been recently put forward [70]. The proposed experiment would employ a millimeter-sized test magnet with mass m=4 mg and a driven source magnet with mass *M* of the same order, levitated and completely shielded from each other in two separate superconducting traps at a relative separation *r* of several millimeters. The experiment would be performed in a low-vibration refrigerator at 300 mK. The expected gravitational acceleration imparted by the driven source mass on the test mass would be in the regime below $10^{-10}$ m/s^2^, as needed to probe MOND-like theories.

By achieving the expected precision of 0.1 $a_{0}$∼$10^{-11}$ m/s^2^ down to even $10^{-3}$$a_{0}$∼$10^{-13}$ m/s^2^, it would be possible to distinguish MOND from Newtonian dynamics at a high confidence level for any separation r≳3 mm and for a large range of masses *M*. Exploring different realizations of the same experiment and providing predictions in terms of both accelerations and velocities will allow to better control systematics in either variable [67,71].

## 5. Conclusions

We have provided an overview of current theoretical and experimental work on levitated micromagnets and discussed current performance as well as open issues and challenges. The extreme sensitivity to forces and accelerations makes levitated magnets, similar to other levitated systems, an excellent candidate for a large number of sensing applications and investigations in the context of quantum and fundamental physics. In addition, the high density of spins provides unique features in the context of magnetometry and the detection of exotic interactions. Owing to the growing number of groups active in this field and the raising interest in levitodynamical systems in general, we expect that a number of the challenges and applications discussed in this review will become a reality in the near future.
